# Application of Nuclear Medicine Liver-Spleen Scan for Evaluation of Littoral Cell Angioma of the Spleen: A Case Report

**DOI:** 10.1177/23247096251350571

**Published:** 2025-06-25

**Authors:** Aren Dermarderosian, Raffi Boghossian, Derek Tai, Javid Sadjadi, Mojtaba Akhtari

**Affiliations:** 1 University of California Los Angeles, USA; 2 Rose and Alex Pilibos Armenian School, Los Angeles, CA, USA; 3 Loma Linda University Medical Center, CA, USA

**Keywords:** littoral cell angioma, nuclear medicine liver-spleen scan, splenic tumor, splenectomy, diagnosis

## Abstract

Littoral cell angioma (LCA) is a rare primary splenic vascular neoplasm originating from the littoral cells of the reticuloendothelial system. Splenectomy is the accepted mode of definitive diagnosis and treatment. With fewer than 200 reported cases, LCA remains poorly understood. Herein, we provide an enhanced insight into its histology and highlight the role of nuclear imaging in aiding LCA diagnosis. A 63-year-old female with a history of stage II multiple myeloma (MM) and rheumatoid arthritis was incidentally found to have a slowly enlarging splenic mass over a 6-year period. Given her candidacy for autologous hematopoietic stem cell transplantation for MM, further evaluation of the splenic lesion was pursued using nuclear medicine (NM) liver-spleen scan, which revealed a photopenic region consistent with a benign hemorrhagic mass. Subsequent splenectomy and histopathological analysis confirmed the diagnosis of LCA, with immunohistochemistry demonstrating CD68+ and CD31+ expression, highlighting LCA’s unique dual histiocytic and endothelial character. This case highlights the diagnostic challenge posed by LCA due to its nonspecific clinical presentation and imaging findings. While splenectomy remains the gold standard for diagnosis, our findings suggest that NM liver-spleen scan imaging may aid in differentiating LCA from malignant splenic masses preoperatively. Furthermore, this case reinforces the association between LCA and hematologic malignancies, supporting the hypothesis that immune dysregulation may play a role in its pathogenesis. This underscores the importance of considering LCA in the differential diagnosis of splenic masses, particularly in cases involving a history of malignancy and/or immune system abnormalities.

## Introduction

Primary splenic neoplasms are rare pathologies with an overall lifetime incidence of 0.1% and can occur as malignant or benign neoplasms.^1^ Malignant splenic tumors include hemangiosarcomas and lymphomas. Rarer malignancies may include fibrosarcoma, leimomyosarcoma, and malignant teratoma; and benign tumors of the spleen include hemangiomas, hamartomas, hemangioendotheliomas, lymphangiomas, and littoral cell angioma (LCA).^2^ With ~150 cases of LCA reported worldwide to date, LCA is a very rare splenic tumor that originates in the red sinus shore cells of the reticuloendothelial system (RES).^3^ Most cases of LCA are benign tumors; however, very few malignant cases have been reported since the condition was described by Falk et al.^4,5^

Given LCA’s relatively recent discovery and low incidence rate, its etiology and pathophysiology remain poorly understood. However, the discovery of its immunophenotypic associations with immune system disorders suggest immune system dysregulation as a driving factor in its etiology.^6-10^

Clinically, most LCA cases are asymptomatic and are discovered incidentally, but occasionally, nonspecific symptoms such as splenomegaly and thrombocytopenia may be present.^2^ As a result, the diagnosis of LCA can be a challenge clinically. In addition, LCA does not have unique radiographic findings, further complicating its diagnosis and differentiation from other solid splenic tumors. However, given the unique functional insight nuclear medicine (NM) liver-spleen scan can offer, it can aid in narrowing the differential diagnosis in the context of splenic masses, as shown in this report.

LCA’s extreme rarity and nonspecific clinical presentation may lead to misdiagnosis or missed LCA detection. Through this case report, we aim to shed more light on LCA and its clinical and histological presentation in order to encourage the consideration of LCA in the differential diagnosis of a splenic mass. In addition, this case report adds to the literature on LCA’s association with hematological malignancies, including multiple myeloma (MM) and further corroborating the possible role of immune dysregulation in the pathogenesis of LCA.

## Case Presentation

**Figure 1.**
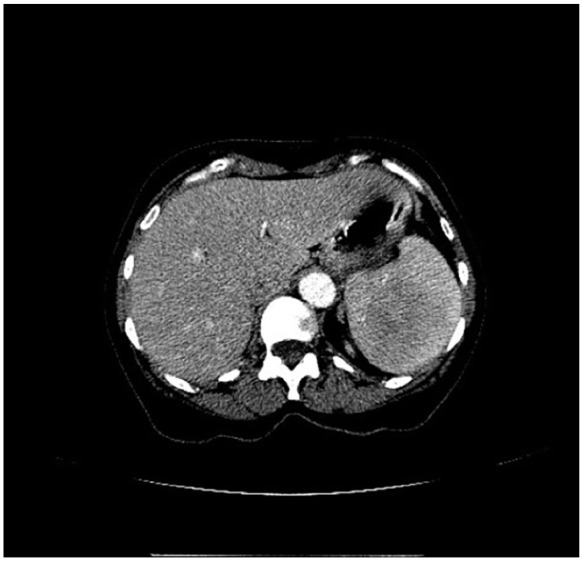
*Transaxial* view of abdominal CT. Indication of enlarging splenic mass within the superior midportion of the spleen.

**Figure 2.**
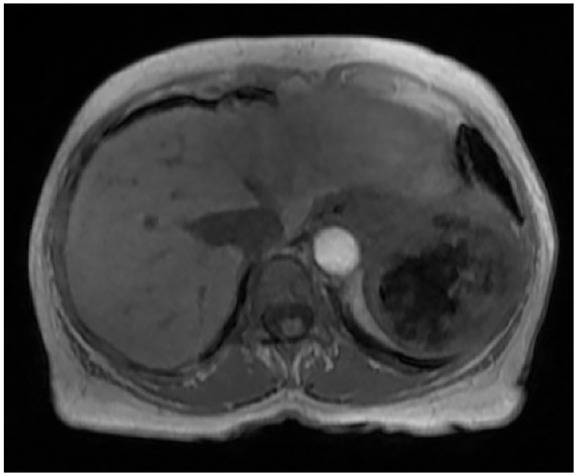
*Transaxial* view of abdominal MRI. The presence of 7.9 × 6.2 × 5.7 cm mass with large necrotic center in the spleen.

A 63-year-old female with a history of light chain, kappa, stage II MM was evaluated for undergoing autologous hematopoietic stem cell transplantation (Auto-HSCT) as part of her MM management. She was found to have a slowly growing splenic lesion, which was first observed on noncontrast Computed tomography (CT) scan 6 years prior to her MM diagnosis although the spleen was read as normal at the time. Consequent imaging performed 5 years after the initial CT scan, showed a splenic lesion measuring 7 × 6.2 cm with the latest CT images showing an enlarging splenic mass within the superior midportion of the spleen (Figure 1). The latest CT findings were followed up with an MRI study, which measured the splenic mass at 7.9 × 6.2 × 5.7 cm with a large necrotic center (Figure 2).

Throughout this period, the patient remained asymptomatic with respect to the splenic mass, with no reports of splenomegaly, abdominal pain, or discomfort. For the MM treatment, first-line therapy with daratumumab, bortezomib, lenalidomide, and dexamethasone was initiated, which was well tolerated. In addition to the MM, she also had a history of rheumatoid arthritis (RA), for which she was treated with methotrexate, and a history of human papilloma virus infection and colonic polyps.

**Figure 3.**
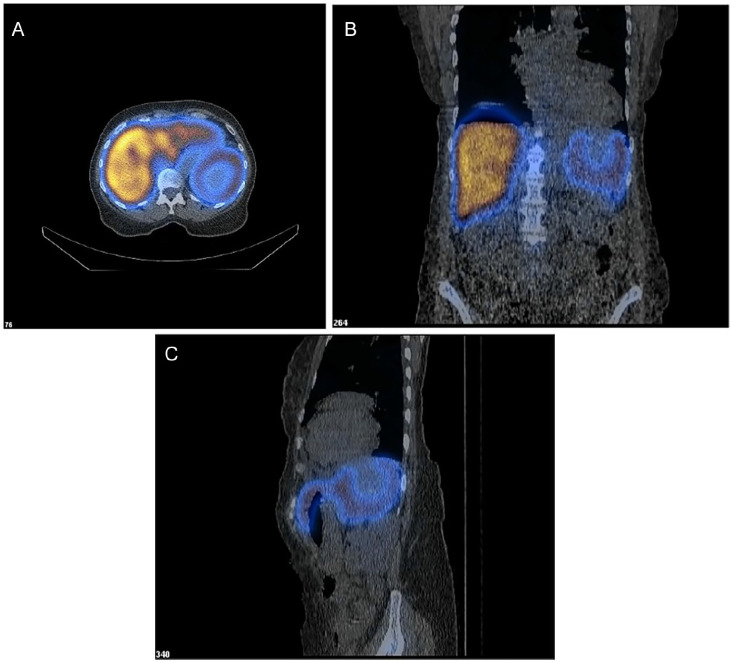
(A) *Transaxial* view from a Tc-99m sulfur colloid liver-spleen scan demonstrating a well-defined photopenic region within the spleen. This area of decreased radiotracer uptake is indicative of a nonfunctioning splenic lesion. Hepatic uptake appears homogeneous. (B) *Coronal view* showing the same photopenic region in the inferior pole of the spleen. The lesion demonstrates no radiotracer accumulation, consistent with the benign nature of the mass. (C) *Sagittal* view further localizes the photopenic defect to the posterior aspect of the spleen. This consistent absence of tracer uptake across multiple planes supports the presence of a space-occupying lesion, which was ultimately diagnosed as a littoral cell angioma following splenectomy.

Since she was being considered for auto HSCT, the decision was made to evaluate the splenic mass despite being asymptomatic before bone marrow transplant (BMT) was initiated. As a result, a NM liver-spleen scan with vascular flow combined with Single Photon Emission Computed Tomography-CT (SPECT-CT) of the abdomen was performed following IV injection of 5.0 mCi Tc-99m. This imaging revealed diffusely increased liver activity with comparatively mild splenic uptake as shown in Figure 3A and B. Additionally, the SPECT-CT imaging showed an ill-defined central photopenic region measuring 4.7 × 3.9 cm in the spleen with surrounding rim uptake and no evidence of colloid shift from the liver to the spleen (Figure 3A–C). These findings suggested the presence of a splenic mass or necrosis within the spleen. Based on the characteristics of the splenic mass and the planned BMT for her MM, a robotic-assisted splenectomy with splenic flexure mobilization was performed by a surgical oncologist. The surgery proceeded uneventfully, and was well tolerated by the patient, who was discharged 2 days postoperatively.

The gross pathological study demonstrated an intact spleen of 267 g and 10.8 × 7.6 × 6.1 cm, with a smooth outer surface glistering with no frank lesions. Sectioning revealed a well circumscribed 9.3 × 7.1 × 6 cm tan-brown to red, soft, and mildly hemorrhagic mass. Moreover, the mass was 1.2 cm from the vascular hilar margins and abutting the splenic capsule, but no involvement was noted. Besides the 9.3 cm mass, no other lesions were appreciated.

**Figure 4.**
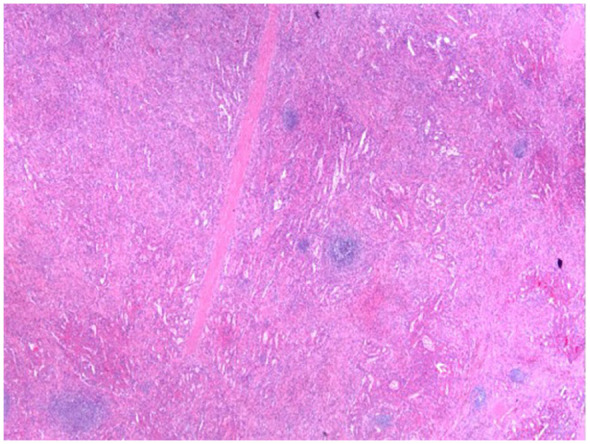
H&E staining of the lesion at low power demonstrates an irregular vascular proliferation expanding the red pulp with preserved intervening white pulp. H&E, hematoxylin and eosin.

**Figure 5.**
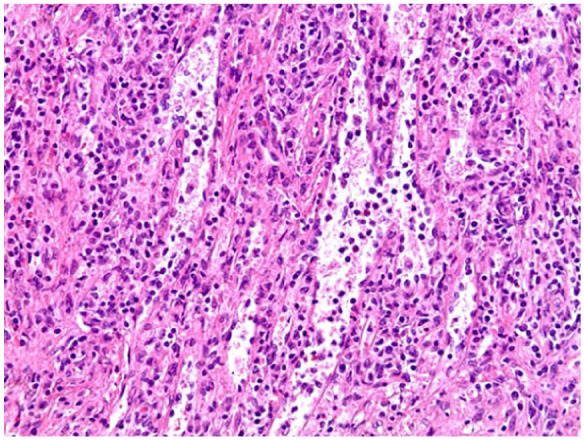
H&E staining of the lesion on high power shows anastomosing channels with tall, hobnailed lining cells that also drop into the vascular lumen. H&E, hematoxylin and eosin.

**Figure 6.**
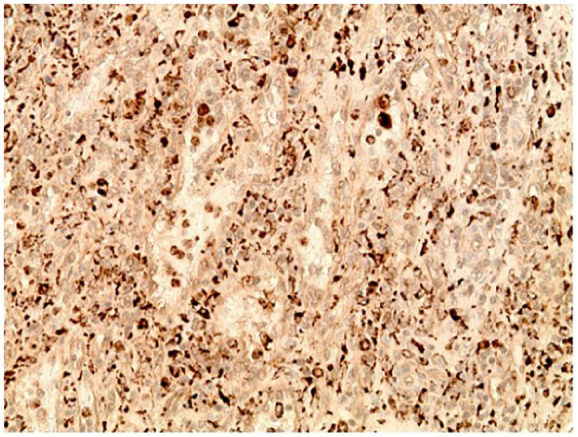
Immunohistochemical staining with CD68 demonstrates positive staining of the lining cells.

**Figure 7.**
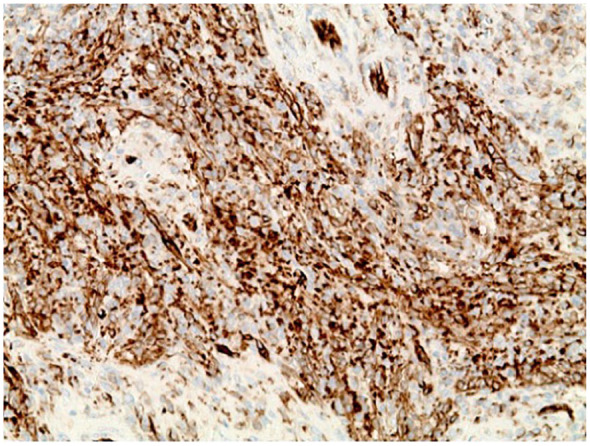
Immunohistochemical staining with CD31 demonstrates positive staining of the lining cells.

Histological studies performed on the lesion showed vascular proliferation within the red pulp of the spleen as seen by an irregular vascular lesion present in the red pulp in Figure 4. Additionally, as seen in Figure 5, the tumor tissue demonstrated anastomosing vascular channels that formed a network with each other that are surrounded by tall endothelial cells without any significant atypia. Furthermore, the immunohistochemical profile of the splenic lesion was determined to be CD68^+^ (Figure 6), CD31^+^ (Figure 7), CD34^−^, and CD8^−^ (Table 1). Ultimately, the histological and immunohistochemical testing results led to the final diagnosis of LCA.

**Table 1.** Results Summary of IHC Testing Performed on the Splenic Lesion.

IHC	Result	Specificity	Interpretation/Comments
CD31	+	Endothelial cells	Expressed in LCA cells, supporting their endothelial differentiation
CD34	−	Endothelial cells	Typically expressed in normal littoral cells; absent in LCA cells
CD68	+	Histiocytic cells	Expressed in LCA cells, indicating histiocytic differentiation
CD8	−	T lymphocytes	Expressed in normal littoral cells; absence supports neoplastic transformation in LCA cells

Abbreviations: IHC, immunohistochemistry; LCA, littoral cell angioma.

## Discussion

**Figure 8.**
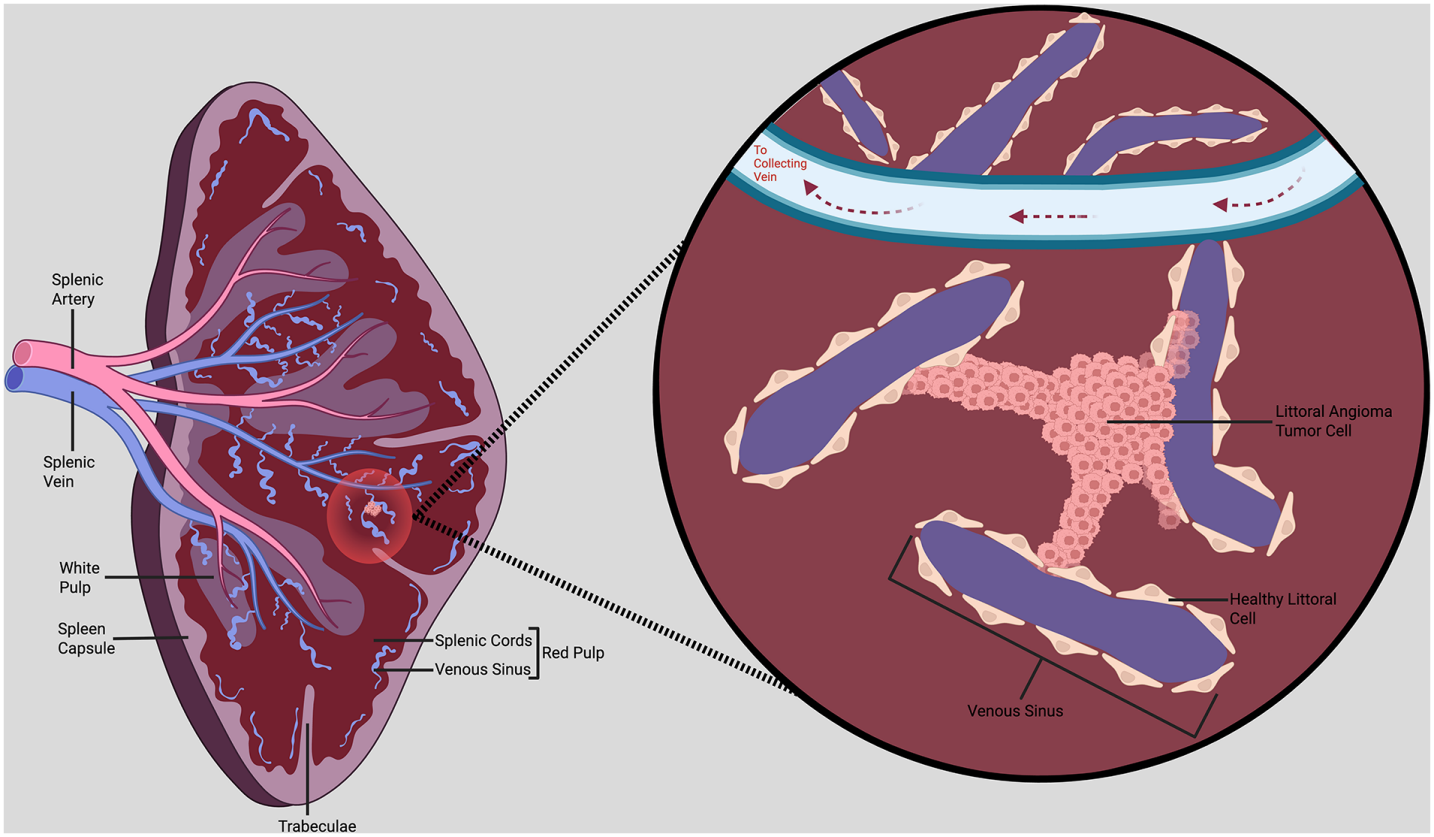
Illustration of littoral cell angioma tumor in the spleen. In healthy splenic tissue, the venous sinuses that make up a part of the red pulp are lined with endothelial littoral cells that act as a physical filter and aid in blood quality maintenance and immune function. In littoral cell angioma, the healthy littoral cells are transformed into clustered tumor cells, disrupting the normal sinusoidal structure and invading into adjacent venous sinuses and other surrounding tissue. (Created in BioRender. Dermarderosian A (2025), https://BioRender.com/b3betjj).

LCA can be classified as a benign primary splenic vascular neoplasm with malignant potential. The littoral cell portion of the condition’s nomenclature reflects the tumor’s origin from the littoral cells that line the venous sinuses in the splenic red pulp. The littoral cells function properly as specialized endothelial-like cells, playing a key role in blood filtration, phagocytosis, and immune surveillance by acting as a physical filter.^11^ However, in LCA, these littoral cells undergo abnormal changes, leading to their uncontrolled proliferation and the formation of vascular tumors in the spleen as shown in Figure 8.

To date, <200 cases of LCA have been reported worldwide, resulting in poor understanding of its etiology.^2^ However, with the increasing coverage of LCA in the literature, various routes of pathobiology have been investigated since its initial report in the literature in 1991.^5,6,8^ Most notably, due to LCA’s close association with autoimmune disorders and other neoplasia, immune system dysregulation potentially has a vital role in the condition’s pathogenesis.^8,10,12^ Some studies have linked LCA with tumor necrosis factor-alpha, Crohn’s disease, and Gaucher’s disease.^12-15^ Furthermore, in a cohort study of LCA patients, 60% of them had co-occurring epithelial or hematologic malignancies.^9^ Additionally, LCA cells express lymphocyte characteristics as evidenced by their expression of CD207 and Cyclin D1 while normal splenic cells do not, which further supports the immune system’s role in LCA’s pathogenesis.^7^

In this case, an immunological component may have had a role in the pathogenesis of LCA given her history of RA and MM diagnosis as immune dysregulation is a hallmark of both conditions.^16,17^ This emphasizes the importance of considering LCA in the differential diagnosis for splenic masses, especially in patients presenting with a history of malignancy or immune system abnormalities.

Close to 55% of LCA cases present asymptomatically, while the rest present with nonspecific symptoms such as diffuse abdominal pain, splenomegaly, anemia, and thrombocytopenia.^18^ Similarly, in this case, the patient was asymptomatic although the splenic mass had been observed 6 years prior to her MM diagnosis and was under observation by CT imaging. However, due to the planned Auto HSCT for her MM, it was imperative to rule out any spleen abnormalities in order to minimize the risk of complications from the Auto HSCT.

A NM liver-spleen scan was performed to evaluate the functional status of the spleen and the RES. The scan revealed a central photopenic region with surrounding rim uptake, a pattern consistent with a hemorrhagic mass, as later confirmed by a hemorrhagic mass in pathology. The observed rim uptake surrounding a central photopenic area suggested a benign splenic lesion, as malignant tumors typically show complete tracer absence due to a lack of RES activity.^19^ To the best of our knowledge, a NM liver-spleen scan has not been used previously in aiding the diagnosis of LCA, and we have shown that a splenic mass due to LCA is detectable by a NM liver-spleen scan and can be useful in providing the functional insight of the spleen and aid in ruling out malignant splenic tumors. However, it should be noted that a definitive diagnosis of LCA cannot be made with a NM liver-spleen scan alone and histopathologic analysis after splenectomy remains the standard of diagnosis.

In an effort to reduce the risk of BMT complication arising from the spleen abnormality, a robotic splenectomy was performed. As with 91% of LCA patients who underwent splenectomy, our patient recovered well, only developing thrombocytosis, which was an expected side effect of the procedure. A single splenic mass of 9.3 cm in diameter was obtained, which is greater than the average diameter of a solitary LCA mass at 6.4 cm.^8^

The splenectomy and the subsequent histology and immunohistochemistry (IHC) served as the main method of a definitive diagnosis. LCA shows a combined pattern of endothelial and histiocytic character as noted by their respective IHC markers.^2,6,8^ Our IHC results for the LCA cells were positive for both endothelial CD31 and histiocytic CD68. This IHC profile highlights one of the main differences between normal splenic littoral cells and their neoplastic counterparts. While normal littoral cells show minimal or variable histiocytic marker expression, LCA cells consistently exhibit dual endothelial and histiocytic differentiation.^5,20^ In addition, CD8 expression in normal littoral cells reflects the organized architecture of the splenic red pulp; consequently, the absence of CD8 expression, as demonstrated in this case, may indicate neoplastic disruption of normal splenic structure.^20^ Ultimately, the histology and the IHC results led to a definitive diagnosis as their descriptions matched the distinct histological features of LCA.

Although LCA has a good prognosis generally, the possibility of malignant transformation remains, especially when the spleen weighs more than 1.5 kg.^18^ This warrants the need for continual surveillance even after a splenectomy given LCA’s association with malignancies.
